# Advancement into the Arctic Region for Bioactive Sponge Secondary Metabolites

**DOI:** 10.3390/md9112423

**Published:** 2011-11-21

**Authors:** Samuel Abbas, Michelle Kelly, John Bowling, James Sims, Amanda Waters, Mark Hamann

**Affiliations:** 1 Department of Pharmacognosy, School of Pharmacy, University of Mississippi, MS 38677, USA; E-Mails: shabbas@olemiss.edu (S.A.); jjbowlin@olemiss.edu (J.B.); jwsims@olemiss.edu (J.S.); alwaters@olemiss.edu (A.W.); 2 National Centre for Aquatic Biodiversity and Biosecurity, National Institute of Water and Atmospheric Research Ltd., Auckland Central 1149, New Zealand; E-Mail: m.kelly@niwa.co.nz

**Keywords:** bioactive molecules, Porifera, sponges, Alaskan Arctic, Aleutian Islands

## Abstract

Porifera have long been a reservoir for the discovery of bioactive compounds and drug discovery. Most research in the area has focused on sponges from tropical and temperate waters, but more recently the focus has shifted to the less accessible colder waters of the Antarctic and, to a lesser extent, the Arctic. The Antarctic region in particular has been a more popular location for natural products discovery and has provided promising candidates for drug development. This article reviews groups of bioactive compounds that have been isolated and reported from the southern reaches of the Arctic Circle, surveys the known sponge diversity present in the Arctic waters, and details a recent sponge collection by our group in the Aleutian Islands, Alaska. The collection has yielded previously undescribed sponge species along with primary activity against opportunistic infectious diseases, malaria, and HCV. The discovery of new sponge species and bioactive crude extracts gives optimism for the isolation of new bioactive compounds from a relatively unexplored source.

## 1. Introduction

Marine sponges have provided a vast resource in the search for bioactive secondary metabolites and potential drug leads. With most research in the area of bioactive sponge metabolites being conducted in temperate and tropical areas and the strong emergence of a myriad of disease resistance, it is becoming increasingly important to survey the fauna of remote regions of the globe for new and replacement drug therapies or compounds with other useful bioactivities. Porifera of cold water environments, including the deep sea and northern polar regions in particular, are marginally known in terms of their faunas, and as such are a relatively untapped resource for scientific discovery. In fact, of all marine natural products described, less than 3% originate from organisms in polar environments [1]. Among the research conducted in polar environments, the majority of compounds isolated and characterized have come from the Antarctic region [1,2]. This article discusses bioactive molecules that have been discovered in the southern reaches of the Arctic Circle and the particular significance of a recent sponge collection conducted in the Aleutian Islands, Alaska. The handful of studies that have been completed in this area are primarily represented by sponges collected in more easily accessible coastal waters of Alaska and off the coast of Sweden and Norway. A potentially fruitful opportunity for new bioactive marine secondary metabolites lies within the cold water regions of the earth, where little, but promising, research has been completed.

Articles addressing cold water bioactive metabolites and the chemical ecology of cold water sponges, particularly the Antarctic region, have been published by our group and others [1–4]. These articles cover some of the compounds listed here and should be consulted for a thorough recording of cold water marine metabolites from various organisms. This work focuses mainly on bioactive sponge metabolites from Alaska and other more northern Arctic cold water regions.

## 2. Secondary Metabolites from Polar Sponges

There are many reasons for the lack of exploration of the metabolites of polar sponges, but the most obvious are the difficulties associated with the access to polar regions and the harsh working environments. Several decades ago the misconception was held that, due to the harshness of the general environment, sponge biodiversity in polar regions would be poor, and competitive pressure to develop chemical defenses were low or non-existent [1]. However, today we know from work in the Antarctic and revised opinions of Arctic benthos that polar regions are rich in sponge diversity [5–7] with comparable species numbers to southern cold-temperate regions [8] and that polar marine invertebrates, including sponges, possess a high number of chemical defenses [9]. Despite the suggestions of high polar sponge biodiversity and thus a potential source for marine drug discovery, less than 100 metabolites from cold water sponges have been described as of 2010, as compared to thousands from temperate and tropical regions [10]. Consequently, extensive sponge surveys in the polar environments for the discovery of new bioactive molecules have merit. The following information reviews the bioactive compounds that have been discovered from marine sponges collected in the southern-most limits of the Arctic Ocean.

### 2.1. Discorhabdin Alkaloids

**Figure 1:**
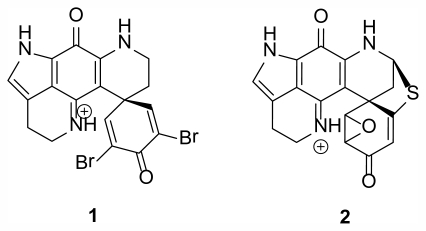
Discorhabdin C (**1**) and Discorhabdin R (**2**); members of the discorhabdin alkaloids, exhibiting a spectrum of bioactivities, isolated in 1986 and 2000, respectively, from New Zealand and Antarctic sponge species of the genus *Latrunculia* [11,15].

One of the major groups of compounds that have been found in polar sponges of the genus *Latrunculia* (Class Demospongiae, Order Poecilosclerida, Family Latrunculiidae) are the discorhabdin alkaloids. Many different derivatives of discorhabdins were originally isolated from Antarctic and New Zealand species of *Latrunculia* yielding an impressive array of activities. Discorhabdin C (**1**) (Figure 1) was first described in 1986 as isolated from the New Zealand species *L*. cf *bocagei* Ridley and Dendy, 1886, and exhibited potent antitumor bioactivity against L1210 tumor cells with an ED_50_ of less than 100 ng/mL [11]. Discorhabdin F [12] was subsequently isolated in 1990 from the Antarctic species *L. biformis* Kirkpatrick, 1907 followed by Discorhabdin G from *L. apicalis* Ridley and Dendy, 1886 [13,14]. Discorhabdin R (**2**) (Figure 1) was isolated in 2000 from an unidentified species of *Latrunculia* collected from Prydz Bay, Antarctica, exhibiting activity similar to other discorhabdins encompassing Gram positive and Gram negative species of bacteria among others [15].

**Figure 2:**
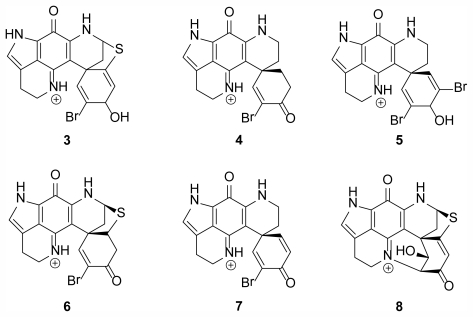
Compounds isolated from a new, undescribed species of *Latrunculia* from the Aleutian Islands. The compounds listed are dihydrodiscorhabdin B (configuration unassigned) (**3**), discorhabdin Y (**4**), dihydrodiscorhabdin C (**5**), discorhabdin A (**6**), discorhabdin E (**7**), discorhabdin L (**8**), and also discorhabdin C (**1**, Figure 1) [4].

In 2009, eight members of the same class of discorhabdin alkaloids, including the two new molecules dihydrodiscorhabdin B (**3**) and discorhabdin Y (**4**), were isolated from members of *Latrunculia* in the Arctic region off the coast of Alaska in the Aleutian Islands (Figure 2) [4]. This was the first report of bioactive compounds from sponges collected in the Alaskan region. The compounds isolated from this new, undescribed species of *Latrunculia*, demonstrated anti-HCV, antimalarial, and antibacterial activities along with two previously described discorhabdins, dihydrodiscorhabdin C (**5**) and discorhabdin A (**6**), showing selective anti-protozoal activity *in vitro* [4]. From this report, compounds **5** and **6**, along with compound **1**, had reported IC_50_ values of 170, 53, and 2800 nM against chloroquine-susceptible *Plasmodium falciparum* and 130, 53, and 2000 nM against chloroquine-resistant *P. falciparum*, respectively. Compounds **5** and **6** were tested *in vivo* using a murine model for antimalarial activity however high levels of toxicity were observed including weight loss, movement reduction, and dehydration. Regardless of these *in vivo* results, the first report of bioactive compounds from an Alaskan sponge being used in an animal model provides promise for future bioactive molecules from the region. Reports suggest that the discorhabdin alkaloids have potential due to their varied biological activities, and new information on the group and the species they have been isolated from [16–19] have been discovered during the past 25 years.

### 2.2. Monanchocidins

**Figure 3:**
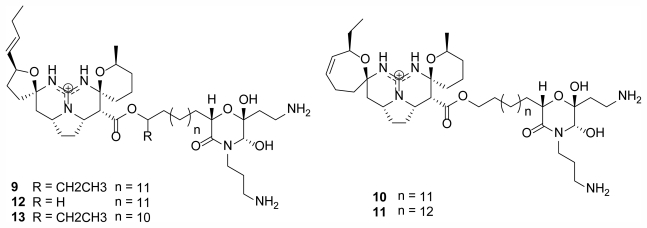
Cytotoxic Monanchocidins A–E (**9**–**13**) isolated from *M. pulchra* [21].

A group of new polycyclic guanidine containing alkaloids were discovered from *Monanchora pulchra* in 2010 and 2011 [20,21] near Urup Island in the southern Sea of Okhotsk at a similar latitude to the discorhabdins in Figure 2. Monanchocidin A–E (**9**–**13**) (Figure 3) demonstrated apoptosis-inducing activity against HL-60 human leukemia cells with 540, 200, 110, 830, and 650 nM respectively [21].

### 2.3. 3-Alkyl Pyridinium Alkaloids

**Figure 4:**
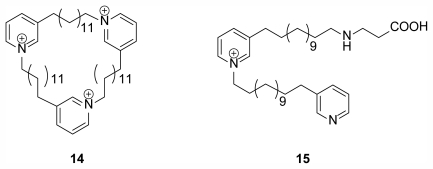
Bioactive compounds viscosamine (**14**) and viscosaline (**15**) isolated from Arctic sponge *Haliclona viscosa* [23].

A series of 3-alkyl pyridinium alkaloids have been isolated from the Arctic sponge *Haliclona* (*Rhizoniera*) *viscosa* (Topsent, 1888) (Class Demospongiae, Order Haplosclerida, Family Chalinidae) with interesting bioactivity including antibacterial, antifungal, cytotoxic, and feeding deterrent effects [22]. Until 2004 *Haliclona* spp. and related genera had only been described from more tropical and temperate environments where they are more abundant and diverse in terms of species. Specimens of *H. viscosa* were collected in Kongsfjorden, an inlet on the west coast of Spitsbergen, an island which is part of the Svalbard Archipelago in the Arctic Ocean. The specimens yielded 2 compounds elucidated as viscosamine (**14**) and viscosaline (**15**) (Figure 4), the first acyclic dimeric 3-alkyl pyridinium alkaloid isolated from nature [23]. Both compounds exhibit antibacterial activity while viscosaline has been reported as a feeding deterrent against the amphipod *Anonyx nugax* and starfish [22].

### 2.4. Diketopiperazines

**Figure 5:**
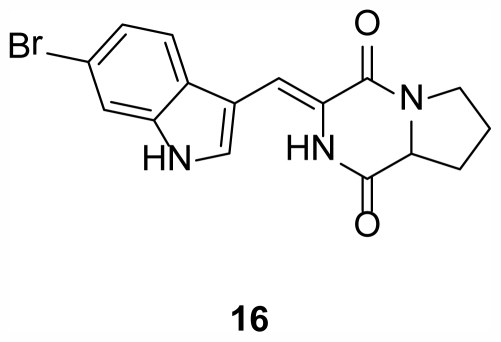
Barettin (**16**), isolated from the sponge *Geodia barretti* collected in the North Sea [24].

Two diketopiperazines, barettin (**16**) (Figure 5) and 8,9-dihydrobarettin were isolated from the sponge *Geodia barretti* Bowerbank, 1858 (Class Demospongiae, Order Astrophorida, Family Geodiidae) in the North Sea off the coast of Sweden [24]. These compounds exhibited extremely interesting non-toxic bioactivity as an antifouling agent, inhibiting the settlement of larvae of the barnacle *Balanus improvisus* and the blue mussel *Mytilus edulis* when mixed with surface coatings.

### 2.5. Polymastiamides

**Figure 6:**
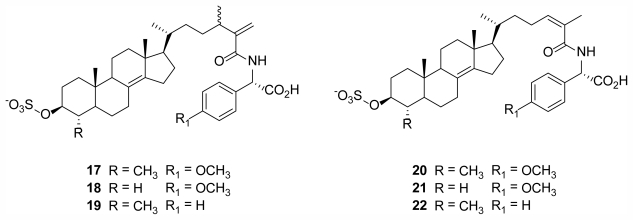
Polymastiamide A, B, E (**17**, **18**, **19**) and C, D, F (**20**, **21**, **22**) isolated from the Norwegian sponge *Polymastia boletiformis* [25,26].

Polymastiamide A (**17**) (Figure 6), the first reported marine natural product derived from a steroid and α-amino acid component, was isolated from the Norwegian sponge *Polymastia boletiformis* (Lamarck, 1815) (Class Demospongiae, Order Hadromerida, Family Polymastiidae), and showed *in vitro* activity against micro-organisms *S. aureus*, *C. albicans*, and *P. ultimum* [25]. Conjugates with similar steroid/amino acid conformations were subsequently isolated from the same sponge species to give polymastiamides B (**18**), C (**20**), D (**21**), E (**19**), and F (**22**) [24] (Figure 6).

### 2.6. Cyclic Peroxides

**Figure 7:**
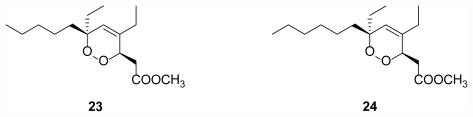
Two cyclic peroxides, **23** and **24**, isolated from the Norwegian sponge *Plakortis simplex* [27].

Another Norwegian sponge, *Plakortis simplex* Schulze, 1880 (Class Demospongiae, Order Homosclerophorida, Family Plakinidae), yielded two new cyclic peroxides (**23**, **24**) (Figure 7) with **24** showing *in vitro* IC_50_ values between 7 and 15 μg/mL for a number of different solid human tumor cell lines [27].

## 3. Recent Collections Around the Aleutian Islands, Alaska

Two recent collection trips were performed in collaboration with the NOAA/AFSC annual groundfish survey. Collections sites in the summer of 2010 were in the western Aleutian Islands between Adak Island and Stalemate Bank. The depths of these collections ranged 60–400 m. Specimens were collected by fishing trawl and were sorted by morphology and immediately frozen at −20 °C. Following the survey samples were shipped frozen for extraction and chemical analysis.

### 3.1. New Arctic Sponges

Our understanding of the sponge fauna of the Aleutian Islands and the Alaskan Arctic is aided by taxonomic literature based on collections principally from the Chukchi and East Siberian Seas, the Aleutian Island chain and Alaskan coastline to the north, Kamchatka Peninsula, Kurile Islands, the Sea of Okhotsk to the west, and the Bering Sea to the north. Unfortunately, unlike the Antarctic region, few large scale collections and taxonomic studies of the overall sponge fauna of this region have been produced [28], and many works are very old and in need of revision. Unfortunately, a full review of the species known from the region has not been possible, but we know from recent studies [29,30] and collections that the sponge demosponge fauna, at least, is diverse and dominated by poecilosclerid taxa.

General literature for the Bering Sea and Alaskan Arctic includes [31–34] for the southwestern Sea of Okhotsk and [35] for the northwest Pacific in general. More recently, Lehnert *et al.* [28] (and in previous works), commenced the documentation of a number of deep and shallow water sponges from the region, many of which were found to be new species. Taxonomic literature from the Gulf of Alaska and British Columbia to the southeast of the Aleutians, and to a lesser extent, the coasts of Washington, Oregon and northern California, are important resources for any work in the Aleutians and Alaskan Arctic. Lambe [36–38], more recently Austin [29], and Austin and Ott [30] provide the best starting points for any taxonomic work in the region. Lee *et al.* [39] on the sponges of California is also particularly relevant.

Of the 93 sponge specimens collected during the 2010 voyage, 6 have been formally identified and two are new, undescribed species. Five out of the 6 specimens are in the Order Poecilosclerida, the most diverse order of demosponges, and typically the most common type of demosponge found in polar regions [6]. The sixth specimen is from the Order Astrophorida, an order of Demospongiae much less common in polar regions than in temperate regions such as around New Zealand [6,8].

**Figure 8:**
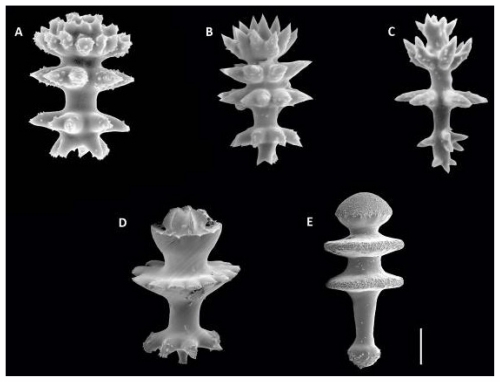
Comparison of the morphology and ornamentation of anisodiscorhabd spicules that distinguish species of *Latrunculia* in the northern Pacific-Arctic region: **(a)** *Latrunculia oparinae* Samaai and Krasokhin, 2002; (**b**) *Latrunculia* n. sp. (dark purple brown hemisphere); (**c**) *Latrunculia austini* Samaai, Gibbons and Kelly, 2006; (**d**) *Latrunculia velera* Lehnert, Stone and Heimler, 2006; (**e**) *Latrunculia occulta* Lehnert, Stone and Heimler, 2006; Scale bar = 10 μm.

Of the 5 poecilosclerid sponges, two were species of *Latrunculia* (Family Latrunculiidae); *L. oparinae* Samaai and Krasokhin, 2002, and the new species detailed in [4]. This genus has presented a major group of compounds, the discorhabdin alkaloids, with a striking array of activities as detailed above. Research on this important group has focused on Antarctic and New Zealand species, but we are now documenting their existence in the North Pacific and southern Arctic regions [11–15]. Prior to this work, *L. oparinae* was known only from the Russian Kurile Islands, in the Sea of Okhotsk, between 127 and 238 m. In the Aleutian Islands, the species is quite common where it is found between 81 and 288 m. The species has a globular shape with tall cylindrical oscules (exhalent structures), and is light olive to khaki green in life. The new, and as yet, undescribed species detailed in [4] is similar to *L. oparinae* but differentiated by color in life. It is dark purple brown, possesses the short flat oscules, and the perfectly hemispherical shape. *L. austini* Samaai, Gibbons and Kelly, 2006, known from the Vancouver Coast of British Columbia, Canada, and further south, is a relatively shallow water species (20–50 m), grayish brown in life, spherical in shape, and with broad crater-like areolate porefields that dominate the sponge surface. *Latrunculia velera* Lehnert, Stone and Heimler, 2006, from the Aleutian Islands, is a dark brown elongate to subglobose sponge with short tiny oscules. The primary feature that differentiates each of these species is the shape and ornamentation of the family-specific microsclere, the anisodiscorhabd (Figure 8. *L. occulta* Lehnert, Stone and Heimler, 2006, also from the Aleutian Islands, is now considered to be a species of the genus *Chondrocladia* (*Meliiderma*) (Order Poecilosclerida, Family Cladorhizidae) [40]. While these species produce an array of different compounds, they are difficult to differentiate at the species level other than by the shape and ornamentation of the family-specific microsclere, the anisodiscorhabd (Figure 8).

Of the 5 poecilosclerid sponges, two were species of *Monanchora* (Family Crambeidae); *M. pulchra* (Lambe, 1895), first described from the Aleutian Islands, and a new, undescribed species, *M*. n. sp. 1 (yellow fan). There are two additional species of this genus known from the Aleutian Islands, Eastern Bering Sea, and the Gulf of Alaska; *M. alaskensis* (Lambe, 1895) and *M. laminachela* (Lehnert, Stone, and Heimler, 2006). The key differences between these 4 species are in the gross morphology of the sponge, the coloration life, the length of the skeleton-forming megascleres, and the length and shape of the microscleres (Table 1).

A new undescribed species of *Guitarra* (Order Poecilosclerida, Family Guitarridae) was amongst the 6 poecilosclerid sponges identified and has been analyzed in greater detail than the other 4 species. The specimen was dredged from a depth of 94 m from the Aleutian Islands, where it was moderately common. In life the sponge forms a massive encrustation with deep cracks outlining polygonal “plates”. The texture is tough, the color in life is peach to orange-yellow. The sponge is characterized by very long megascleres (oxeas 580–600 μm long) and three forms of microscleres (biplacochelae, c. 47 μm long, in addition to the usual placochelae, c. 47 μm long, and small, spiny bipocillae and anisobipocillae, c. 15 μm long). The most closely comparable species to *Guitarra* n. sp. is *G. abbotti* Lee, 1987, from Cordell Bank, northern California. *Guitarra abbotti* also has the unusual biplacochelae in addition to the usual oxeas, placochelae, and bipocillae microscleres, but these differ considerably in length from those in *Guitarra* n. sp. The oxeas of *G. abbotti* are half the length (330 μm) of those in *Guitarra* n. sp., and there are two sizes of placochelae in the former species, the larger of which is twice the length of those in *Guitarra* n. sp. (83 μm long) with the smaller size being 37 μm long. The biplacochelae of *G. abbotti* are slightly smaller (36 μm) than those in *Guitarra* n. sp, and the bipocillae are half the length (7 μm) of those in the new species.

The single astrophorid species is *Poecillastra rickettsi* de Laubenfels, 1930 (Family Pachastrellidae), first described from northern California.

### 3.2. Isolation and Elucidation Efforts

Each of the 93 sponge samples were initially processed by extracting 50 g portions (wet) with ethanol. The resulting extract was submitted for opportunistic infections, antimalarial, and antiHCV assays. For more rapid results, each extract was screened using a disc diffusion assay against *B. cereus*. Extracts were tested at 1 mg/mL concentrations with kanamycin used as a positive control.

The new species of *Guitarra* was examined because of initial activity from the disc diffusion assay against *B. cereus*. The complete 1 kg (wet wt.) specimen was extracted with ethyl acetate to yield 11 g. The resulting extract was subject to a silica flash column and the activity eluted from a fraction of 1:1 hexane and ethyl acetate. The resulting fraction (483 mg) was subject to separation using 3 cm diameter LH20 column and a 1:1 mixture of dichloromethane and methanol. The activity was traced to a group of fractions that were combined then subjected to further separation on a Waters Delta HPLC system equipped with an analytical scale UV detector set to 280 nm and using a 4.6 × 150 mm Phenomenex Luna Silica column eluted with a linear gradient from 100% pentane to 100% dichloromethane over 75 min. The HPLC separation yielded sub-miligram amounts of two pure compounds that were identified as 4-hydroxybenzaldehyde and indole-3-carboxaldehyde based on comparison of standards with ^1^H NMR and GC-MS results (supplemental information).

### 3.3. Bioactivity

**Figure 9:**
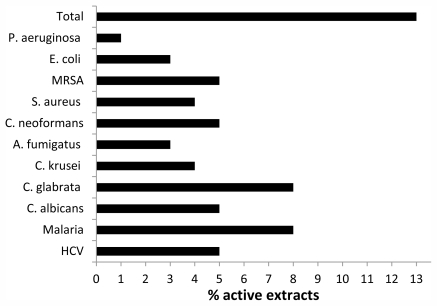
The percent of crude extracts of 93 Alaskan sponge samples that yielded significant activity (greater than 50% inhibition *in vitro*) against different opportunistic infections (*Candida albicans*, *Candida glabrata*, *Candida krusei*, *Aspergillus fumigatus*, *Cryptococcus neoformans*, *Staphylococcus aureus*, Methicillin-resistant *Staphylococcus aureus*, *Escherichia coli*, *Pseudomonas aeruginosa*), malaria, and hepatitis C virus (HCV)(methods described in [4]).

An initial *in vitro* screen of 93 crude sponge extracts revealed bioactivity against many microorganisms associated with opportunistic infections (Figure 9), indicating a broader than expected activity against a small sample set of different microorganisms. The extracts were also screened against *Plasmodium falciparum* (malaria) and HCV resulting in the identification of several samples with significant activity.

The IC_50_ values for active sponge extracts ranged from 200 to 5000 ng/mL against the different opportunistic infections (ciprofloxacin and amphotericin B as controls) and antimalarial IC_50_ ranged between 500 and 2200 ng/mL (chloroquine and artemisinin as controls); anti-HCV IC_50_ values were not determined. Many of the active samples are currently being examined for their active components and will be reported in due time. The initial screen for activity combined with the evidence of interesting taxonomic classification highlights the potential of the polar region for new bioactive compounds.

Antibacterial activity for *Bacillus cereus* guided the isolation and lead to the elucidation of two known aldehydes mentioned previously identified from the new sponge species of the genus *Guitarra*. Indole-3-carboxaldehyde was isolated previously from marine *Pseudomonas* species [41]. Using purchased standards of these molecules, the original bioactivity of the crude extract was unable to be reproduced. In spite of this result, 4-hydroxybenzaldehyde has been reported as active against some bacterial and yeast species [42]. Interestingly, it has also been isolated from a perennial saprophytic herb *Gastrodia elata* and demonstrated unique bioactivity. Assays performed *in vivo* using rats proved to give 4-hydroxybenzaldehyde anticonvulsive and antiepileptic properties [43].

## 4. Conclusions

**Figure 10:**
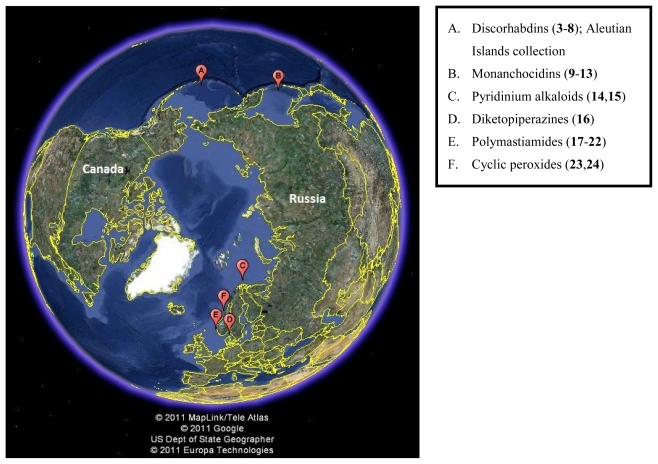
A view of the earth directly over the North Pole which indicates the collection sites that yielded the bioactive metabolites presented in this review. The following image was created using Google Earth (version 6.1.0.5001).

With roughly 70% of the earth covered with ocean, marine sources provide an enormous source for scientific research and discovery. Numerous collections conducted for over half a century to collect sponges in particular for their bioactive secondary metabolites have been carried out in many cold temperate, temperate, tropical, and subtropical locations across the Atlantic and Pacific Oceans and in other parts of the globe. These efforts and their results are thoroughly described in a series of reviews [44]. By contrast, dedicated collections of sponges to specifically isolate and characterize metabolites from sponges in the Arctic and Alaskan regions have been few and far between as illustrated in Figure 10, and yet the biodiversity of the Gulf of Alaska and British Columbia in particular, is known to be considerable. The discovery of new species with diverse bioactivity as indicated by the initial screen of the 2010 collection of Aleutian sponges and previous collections made by our group in the general area bodes well for the future of new and valuable bioactive compounds.

## Supplementary Material



## Figures and Tables

**Table 1:** Key differences between species of *Monanchora* (Order Poecilosclerida, Family Crambeidae) in the Aleutians-Arctic region.

	*Monanchora pulchra*	*Monanchora* n. sp. 1 (yellow fan)	*Monanchora alaskensis*	*Monanchora laminachela*
**shape**	ramose fan	fan	short flabby	subglobular
**color in life**	red	yellow	brown	yellow
**styles (interior) (μm)**	1100	480–510	262	840–1170
**styles (dermal) (μm)**	176–478	200–250	144	350–395
**microscleres 1 (μm)**	19	30–35	91	22–25
**microscleres 2 (μm)**	13	20	32	19–23
