# Supplementary material for: Advancement into the Arctic Region for Bioactive Sponge Secondary Metabolites
Source: Mar Drugs. 2011 Nov 21;9(11):2423–37. doi: 10.3390/md9112423 (PMC3229243; doi:10.3390/md9112423)

Supplemental Information

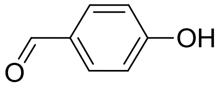

20
4-hydroxybenzaldehyde
Chemical Formula: $C_7H_6O_2$
Exact Mass: 122.0

Predicted ^{1}H NMR δ: 9.88 (s, 1H), 7.82 (d, J = 7.5, 2H), 6.89 (d, J = 7.5, 2H), 5.35 (bs, 1H)

Experimental ^{1}H NMR δ : (400 MHz, $CDCl_3$) 9.88 (s, 1H), 7.81 (d, J = 8.3 Hz, 2H), 6.95 (d, J = 8.3 Hz, 2H), 5.41 (bs,1H)

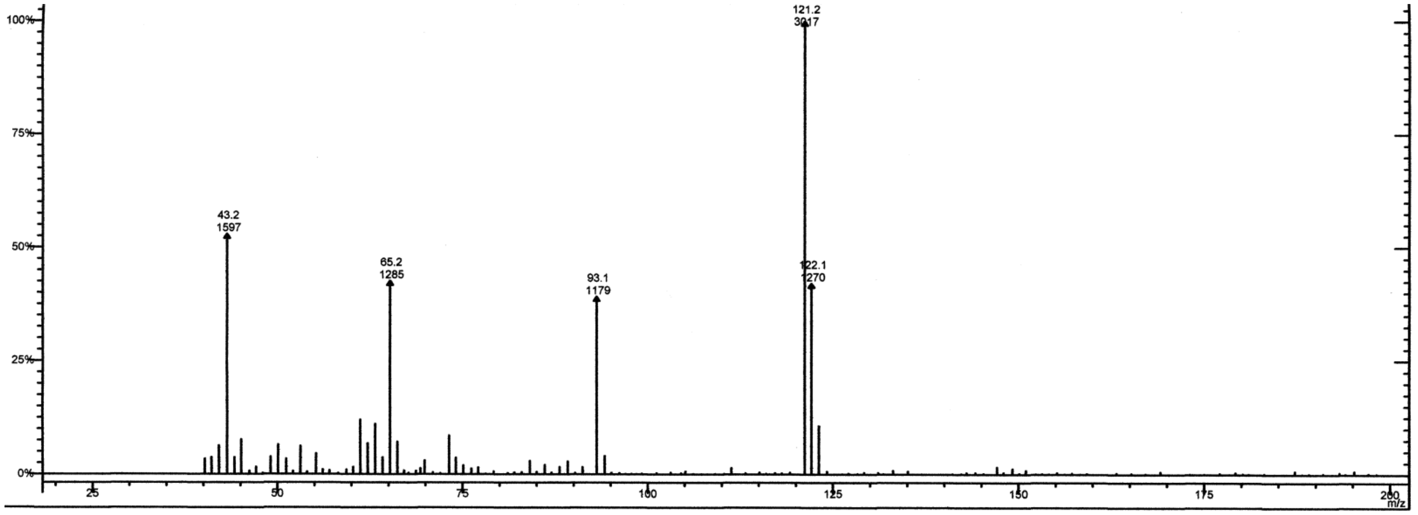

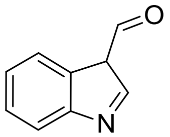

21
indole-3-carboxyaldehyde
Chemical Formula: C_9H_7NO
Exact Mass: 145.1

Predicted ^{1}H NMR δ: 9.72 (d, J = 6.2, 1H), 7.50 (d, J = 6.2, 1H), 7.33 (dd, J = 7.5, 1H), 7.26 (dd, J = 7.5, 1H), 7.23 (d, J = 7.5, 1H), 7.01 (d, J = 7.5, 1H) 3.6 (dd, J = 7, 1H)

Experimental ^{1}H NMR δ : (400 MHz, $CDCl_3$) 10.0 (s, 1H), 8.33 (m, 1H) 7.85 (bd, 1H) 7.45 (m, 1H) 7.33 (m, 2H) 1.25 (s, 1H)

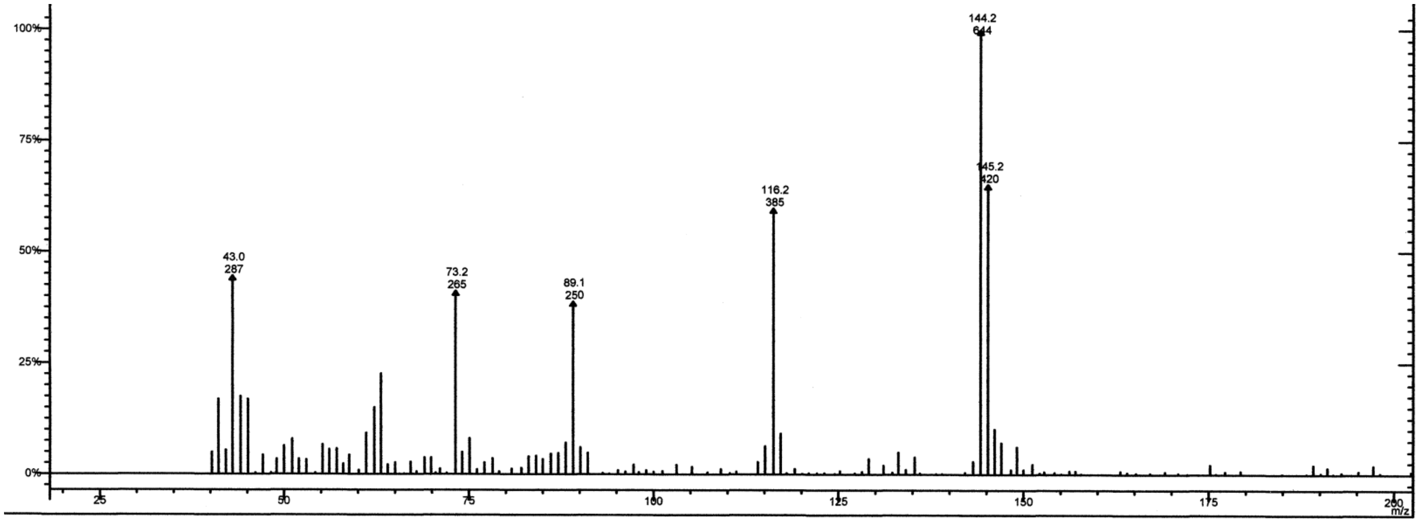

Supplement: Supplementary file 1 [file marinedrugs-09-02423-s001.pdf]
